# Applications of technology in the assessment and treatment of cannabis use disorder

**DOI:** 10.3389/fpsyt.2022.1035345

**Published:** 2022-10-19

**Authors:** Christina A. Brezing, Frances R. Levin

**Affiliations:** ^1^Division on Substance Use Disorders, New York State Psychiatric Institute, New York, NY, United States; ^2^Department of Psychiatry, Columbia University Irving Medical Center, New York, NY, United States

**Keywords:** cannabis, cannabis use disorder (CUD), technology, ecological momentary assessment, treatment, assessment

## Abstract

Cannabis use and Cannabis Use Disorder (CUD) have been increasing. There are no FDA approved medications and evidence-based psychotherapy is limited by insufficient providers, serving very few patients effectively. The lack of resources for prevention and treatment of CUD has resulted in a significant gap between the need for services and access to treatment. The creation of a scalable system to prevent, screen, refer and provide treatment for a chronic, relapsing diagnosis like CUD could be achieved through the application of technology. Many studies have utilized ecological momentary assessments (EMA) in treatment seeking and non-treatment seeking cannabis users. EMA allows for repeated, intensive, longitudinal data collection *in vivo*. EMA has been studied in cannabis use and its association with affect, craving, withdrawal, other substances, impulsivity, and interpersonal behaviors. EMA has the potential to serve as a valuable monitoring tool in prevention, screening, and treatment for CUD. Research has also focused on the development of internet and application-based treatments for CUD, including a currently available prescription digital therapeutic. Treatment options have expanded to more broadly incorporate telehealth as an option for CUD treatment with broad acceptance and change in regulation following the COVID-19 pandemic. While technology has limitations, including cost, privacy concerns, and issues with engagement, it will be a necessary medium to meet societal health needs as a consequence of an ever-changing cannabis regulatory landscape. Future work should focus on improving existing platforms while ethically incorporating other functions (e.g., sensors) to optimize a public and clinical health approach to CUD.

## Introduction

Cannabis use disorder (CUD) is a public health problem associated with psychiatric and medical morbidity (1, 2), poor performance (3), and decreased quality of life (4). In 2020, 14.2 million people aged 12 years and older met criteria for CUD (5). Despite the large number of patients with CUD, we have limited treatments. There are no Food and Drug Administration (FDA) approved medications; however, many pharmacological trials have been completed with some support for off-label usage of certain medications, the overall evidence to date is incomplete and does not provide definitive guidance (4, 6). Evidence-based psychotherapies are the primary intervention (7). There is a notable treatment gap for CUD, in that a minority of patients who would benefit from specialized treatment do not have access (5). Additional factors that contribute to this include stigma, poorly disseminated information regarding where to access treatment, misinformation about the abuse potential and safety of cannabis, geographic barriers and transportation limitations, a non-patient centered treatment system (e.g.: schedule based on provider not patient), limited integration and lack of referrals within the larger healthcare system, lack of insurance coverage for services, lack of perceived need for specialty care by patients and other providers, and concerns for privacy and confidentiality (8).

Simultaneously, the majority of adults living in the U.S. has access to home broadband internet (77%), and a smartphone (85%), which has been increasing year over year (9). Given this landscape, applying technology in the assessment and treatment of CUD has the potential to reach patients at scale with effective treatments by building a technology-enabled infrastructure to optimally prevent, screen, treat, and minimize the consequences of cannabis use and the development of CUD. CUD is an especially promising target for digital assessments and interventions. The chronic nature of the disorder requires a treatment paradigm that can increase patient and provider awareness through monitoring, initiate abstinence, prevent relapse, provide maintenance support, and interventions if relapse occurs to reinitiate abstinence. This model requires proactive and longitudinal assessment and treatment that is difficult to provide in traditional brick and mortar clinics. Clinical research in the application of technology to the assessment and treatment of CUD is in its incipient stages, though important work has been done demonstrating clear feasibility, acceptability, and clinical utility of many modalities. This brief review will cover research of technology in (1) the assessment of cannabis use and other key factors in CUD, (2) the treatment of CUD, (3) limitations, and (4) future directions.

## Use of technology in the assessment of cannabis use and cannabis use disorder

Successful public health campaigns must appropriately screen, monitor, accurately assess, and then either prevent the development of CUD or refer for services (10). The use of technology in the form of screening assessments provided through digital platforms would be the first step in this systematic public health approach to cannabis (11). Ecological momentary assessments (EMA) can prospectively capture behavior, emotion, or cognitive functioning in close to real-time through scheduled, random, or self-initiated prompts longitudinally on a smartphone. The *in-vivo* assessment minimizes the impact of memory degradation and recall biases and provides high real-world validity (12, 13). EMA, its frequency, and duration of collection can be highly tailored to the population of interest. EMA has been shown to be clearly feasible and acceptable in individuals who use cannabis, including those with co-occurring disorders. Individuals who use cannabis or have CUD have high average completion rates of prompts of 85–86%, with lower rates of completion of ~75% in populations with notable disability and polysubstance use (14, 15). Studies using EMA to assess individuals using cannabis or with CUD have been completed in multiple states (15). The lack of geographical limits and the increased representation from historically harder to reach groups opens the door for both national and international projects. As an example, nationally there is an opportunity to elucidate changes in health with changes in state policy related to recreational and medical cannabis legalization (15). EMA has the added clinical benefit of improving self-awareness in participants who report finding EMA “helpful” in brining attention and greater agency to their cannabis use (15, 16). EMA has been used in both treatment seeking and non-treatment seeking individuals using cannabis (16), and to assess use of multiple substances that may be related to use of cannabis, including nicotine, and alcohol, providing valuable information on the complicated nature of co-use and impact on cognitive functioning (17–19). EMA has been implemented on study provided smart phones and on participants' own phones. The former ensures equity of access and user experience; the later providing for greater generalizability for use in a real-world population.

Research has utilized EMA to assess existing policies and programs for cannabis use prevention and mental health support in addition to assessing participants' experiences with mental health and behaviors (20). EMA has demonstrated how cannabis use may mediate suicidal ideation in youth in addition to identifying that bullying and being a gender minority resulted in increased risk (20). In addition to looking at suicidal ideation and stressful experiences (e.g., bullying), multiple studies of EMA and cannabis use have explored positive and negative affect and craving as it relates to risk of use, demonstrating a mixed and inconsistent relationship, with some studies finding cannabis serves more as positive reinforcement and in other studies as a negative reinforcement with overall severity of CUD driving sharper increases in positive reinforcement and craving (16, 21, 22). Other research has assessed anxiety, craving, social factors (e.g., peers using cannabis), and withdrawal symptoms in individuals with regular cannabis use and CUD to see how they are related to cannabis cessation or continued use (23–25), finding that anxiety, craving, and environmental factors most greatly influenced use of cannabis. Other studies have assessed impulsivity and interpersonal hostility as mediators of cannabis use, finding that cannabis use increased impulsivity and hostility (26, 27). These findings suggest that factors driving cannabis use, including affect and craving, are dynamic, transient, and variable neurobiological processes within individuals and may underlie cannabis use problems (28).

A recent study collected EMA data in adolescents while also capturing objective data from smartphones to improve data quality and enhance data fidelity including use of the smartphone camera to capture and provide photographs of participant identification to confirm eligibility, activity tracking on the phone to assess usage and screentime, accelerometer data to estimate physical activity, and the smartphone's global positioning system (GPS) to assist with geolocation and confirmation of school attendance (20). Many studies depend solely on self-report of outcomes of interest, ultimately limiting conclusions and understanding of biological and environmental factors that are not captured (29). Future studies that use EMA should build off the work done to date and combine other technologies that can also collect passive, objective data, such as can be done with the use of sensors, to capture these complex intra-individual processes in conjunction with biologics and cannabinoid testing to confirm use.

To date, no study that these authors are aware has been completed and published utilizing EMA in a clinical treatment trial for CUD as the primary outcome, though current studies are underway [(16); NCT05273567; NCT05322941]. Given that EMA has been shown to be superior to global self-report, fixed time assessment measures, and retrospective self-report, making use of EMA to assess treatment outcomes in CUD treatment trials will be valuable in improving data quality and limiting recall bias inherent to traditional primary endpoints (Timeline Followback) (30).

## Use of technology in the treatment of cannabis use disorder

Clinical research, including randomized controlled trials, utilizing technological platforms in the treatment of CUD, as part of substance use disorder treatment more broadly, began in the late 1990's and early 2000's with the emergence of the first wave of digital therapeutics and coincided with a global interest and increased utilization of the internet to fulfill new functions. These internet-based programs offered an opportunity to deliver high fidelity interventions at scale (31). Clinical research of face-to-face, manualized psychotherapies for CUD, such as Motivational Interviewing, Cognitive Behavioral Therapy and Contingency Management, emerged as clearly efficacious and made use of tangible and teachable skills and behavioral theories, such as mindfulness, breathing and relaxation exercises, and reward incentives, while adhering to an educational framework that explained and practiced through homework behavioral activation, cognitive restructuring, goal-setting, and strategies for coping. These treatments translated well to online materials and internet platforms (32). Early versions of these internet- based programs for CUD and other substance use disorders, such as the Therapeutic Education System (TES) (33) and CBT4CBT (34) were delivered *via* web-pages and accessible through internet connection on computers. These treatments consistently demonstrated improvements in clinical outcomes and increased acquisition of skills for patients with substance use disorders including CUD in large, randomized, controlled trials (33, 35). Additionally, studies found that these technology-based interventions are superior to no treatment and are non-inferior to in-person psychotherapy (36–38).

The internet-based treatment programs for CUD and other substance use disorders provided the foundation for a second and current wave of technological interventions for addiction. Developing in parallel with the ecosystem allowing for internet access through smartphones and tablets, these treatments were formatted into downloadable software in the form of applications (“apps”). Apps have the benefit of interfacing with and accessing features of smartphones not typically used in desktop computers such as cameras, sensors, and location through GPS (39). They also allow for continuous communication, including when patients are not currently using the application by enabling prompts through push notifications (39). Some application functions may work offline allowing for access in the absence of an internet connection (39). Mobile applications for substance use disorders such as CUD most notably include the Food and Drug Administration (FDA)- approved prescription digital therapeutic reSET^TM^, developed by Pear Therapeutics Inc. (US) for the treatment of substance use disorders. reSET^TM^ provides application-accessible CBT-based treatment modules. It has functions that include health education, identification of triggers, individualized strategies and skills to address cravings and high-risk situations, assessments and feedback of the patient's acquisition of therapeutic principles, and a clinician dashboard to assist in overseeing patient progress during the 12-week prescription-required program. During this time patients also monitor and report on substance use, cravings, or triggers, while the healthcare provided can input urine toxicology results and record current medications. This comprehensive prescription digital therapeutic has been shown to effectively support abstinence and reduce the risk of relapse. reSET^TM^ was designed to be used in conjunction with in-person sessions with prescribers in the treatment of substance use disorders including CUD, allowing for a hybrid application of technology. This prescription digital therapeutic requires a prescription by a clinician to access and download the platform. Unlike health and wellness applications that patients can access directly, prescription digital therapeutics are rigorously evaluated for safety and effectiveness in randomized controlled trials and must meet authorization standards by the U.S. Food and Drug Administration (FDA). They are subject to post-marketing requirements that are similar to regulated pharmaceuticals. These FDA approved software treatment products also include stringent security and privacy controls which is highly important given the nature of the health information collected (40).

Web-based programs, such as TES and CBT4CBT, and prescription digital therapeutics like reSET^TM^, are treatments that transdiagnostically address substance use disorders including CUD. Multiple rigorous randomized, controlled trials have also been completed using self-guided (41, 42), clinician guided, or chat-supported (43, 44) web-based treatment programs specific to CUD. These studies found internet-based programs to be effective alternatives for lower severity and less complicated patients or potentially helpful supplemental resources in reducing cannabis use and symptoms if not adequate as stand-alone treatments for abstinence.

The use of telehealth for the treatment of CUD has never been more important or more widely utilized. Telehealth is the remote delivery of healthcare using telecommunications technology, most commonly by video conferencing (45). Prior to the COVID-19 pandemic, there were notable financial, legal, and regulatory barriers that prevented widespread adoption of delivering treatment for substance use disorders remotely facilitated by technology (46). In March 2020, utilization of telehealth in the US increased 154% in <1 month (47), fueled by policy changes that improved provider payments for telehealth, permitted interstate treatment, authorized multiple types of providers to perform telehealth services, reduced or waived cost-sharing for patients, allowed for virtual visits to be conducted from the patient's home, rather than a clinic, and granted widespread permission to federally qualified health centers or rural health clinics to offer telehealth services (48). Prescribers quickly adapted to the new environment in utilizing home-based telehealth as a primary means of providing treatment. This means of providing treatment is likely to continue to be a mainstay option for patients to access specialized treatment for CUD, as both providers and patients find it satisfying and convenient (49, 50).

## Limitations and considerations

While the assessment and treatment of CUD with technology brings many promises, there are some limitations and considerations that will need to be addressed. Privacy, anonymity when possible, and data sovereignty at the level of the individual must be primary goals when considering the use of technology and its ability to prevent and treat CUD. Confidentiality must be protected through the use of passwords, data de -identification, and encryption. Informed consent should be clear and explicitly presented with regards to actively and passively collected data. Options to drop-out and stop data acquisition should be presented and easily fulfilled if desired. Research and treatment with technology will need to ethically balance participant confidentiality with data quality and reach of scientific understanding (51). Internet and smartphone inequity is an additional barrier, particularly for individuals who are older, have unstable housing, and live in countries with inadequate infrastructure to support technology dependent services, such as in developing nations and more rural areas (11, 52).

Despite the availability of existing applications and a commercially available prescription digital therapeutic, the overall impact on assessing and treating CUD is low. Factors contributing to low penetration of technological tools for CUD include lack of awareness of these mobile health treatments by providers and patients, limited adoption by patients and providers due to factors outlined above, and low patient engagement, usage, and adherence, particularly over time (53). In the case of prescription digital therapeutics, prescribers may not be familiar, comfortable, or competent in deploying these therapeutics. Prescribing a non-medication treatment to a patient may be novel for many clinicians since it is operationally distinct from referring a patient for psychotherapy. Most clinicians are not trained in the use of technology for treatment, outside e-prescribing and documentation in electronic health records, which may slow adoption of new modalities and subsequent uptake by patients. Healthcare and psychology graduate school programs should add training in digital technologies for CUD to facilitate patient access.

Engagement and completion rates have been low to moderate with digital interventions for CUD. Engagement may be improved by integrating content, language, interfaces, delivery systems and rewards that are more salient and specific to the individual. For example, applications for adolescents should include elements that are more relevant to this age group (54). Design of these interventions must take into account key, qualitative differences in the experience of the patient and clinician (55). Research should consider incorporating social media and gaming that could be applied to CUD assessments and interventions to improve engagement, particularly in youth.

Finally, initial costs of purchasing, learning, and implementing new digital assessments and treatments takes time and financial investment. There are limited codes for payer reimbursement for reviewing a technological assessment or utilizing a prescription digital therapeutic. The current system will need to adapt to provide compensation and coverage for these services to further adoption.

## Future directions

Future research, screening, prevention, and treatment for CUD should continue to build upon the strong work completed today. The use of EMA should expand to build toward not just in-depth assessment but ecological momentary interventions (EMI) that can be specific and appropriate (56, 57). Machine learning has already been able to predict the folding process of 200+ million proteins based on their amino acid sequencing (58). Applying machine learning to predict risk for the development of CUD or provide informed interventions to prevent engagement in cannabis use in real-time while simultaneously providing access to digital services (59, 60).

Internationally there have been increases in cannabis consumption across ages (61). Assessments and treatments delivered through technological platforms can easily be translated into different languages. Mobile health interventions targeting cannabis have been applied to helping pregnant people (62), individuals with comorbid psychiatric disorders like psychosis (63), students in schools (20), incarcerated offenders (64), co-occurring chronic pain and opioid use (15), individuals with cannabis and other substance co-use (18, 65), and racially diverse populations (66). Future work can modify existing applications or build new, custom platforms to improve and expand the reach of these assessments and treatments to support diverse populations through socially and culturally competent means. Targeted interventions can prioritize at risk groups. While some interventions to have allowed for a patient-clinician interface, future versions should expand the support network to include therapists, family-members, peers, and other stakeholders to provide input and resources toward the prevention or treatment of cannabis use and CUD.

As the field matures, we anticipate that technology will be ubiquitous in research and clinical care of CUD. While work to date has focused on patients or providers actively inputting their observations, subjective accounts may be best utilized in a limited fashion. Ideal characteristics of digital tools of a chronic, relapsing disorder like CUD allow for continuous monitoring, are unobtrusive and create a lower user burden, and allow for remote acquisition of data in a naturalistic setting. Future work should focus on these goals and optimize for them by expanding into other areas such as social media, augmented and virtual reality, and sensors.

## Conclusions

Effective applications of technology have the potential to address the gap in treatment and prevent the development of CUD in high-risk groups. EMA and web and application- based treatments have been studied most extensively and have the greatest data supporting their feasibility, acceptability, and efficacy for CUD, culminating in an FDA approved prescription digital therapeutic. Current clinical treatment should utilize these tools and future research should expand to explore the use of other technology for CUD while improving existing applications. As with any new technology, attention must be given to its limitations to thoughtfully and effectively derive the greatest benefit.

## Author contributions

CB conceptualized the paper and wrote the first draft. CB and FL discussed approach and outline. FL reviewed and provided edits and revisions. All authors incorporated journal-provided reviews and edits.

## Funding

CB was supported by NIDA funded K23DA045080 and R21DA055835, and the Columbia University Vagelos College of Physicians and Doris Duke Charitable Foundation Fund to Retain Clinical Scientists. FL has a NIDA funded K24DA029647.

## Conflict of interest

The authors declare that the paper was written in the absence of any commercial or financial relationships that could be construed as a potential conflict of interest.

## Publisher's note

All claims expressed in this article are solely those of the authors and do not necessarily represent those of their affiliated organizations, or those of the publisher, the editors and the reviewers. Any product that may be evaluated in this article, or claim that may be made by its manufacturer, is not guaranteed or endorsed by the publisher.
